# Invasive Group A* Streptococcus* infections in children during the post-pandemic period: results from a multicenter study in Italy

**DOI:** 10.1186/s13052-025-02103-7

**Published:** 2025-11-28

**Authors:** Elena Chiappini, Marco Renni, Maia De Luca, Samantha Bosis, Silvia Garazzino, Laura Dotta, Raffaele Badolato, Federica Zallocco, Daniele Zama, Antonella Frassanitto, Ilaria Liguoro, Danilo Buonsenso, Claudia Colomba, Lorenza Romani, Giulia Lorenzetti, Federica Ceroni, Marco Denina, Nicolò Monti, Catiuscia Lisi, Luisa Galli, Giangiacomo Nicolini, Guido Castelli Gattinara, Andrea Lo Vecchio, Italian Society of Pediatric Infectious Diseases (SITIP)

**Affiliations:** 1 Infectious Diseases Unit, Meyer Children’s Hospital IRCCS, Florence, Italy; 2 Department of Health Sciences, University of Florence, Florence, Italy; 3 Postgraduate School of Pediatrics, Department of Health Sciences, University of Florence, Florence, Italy; 4 Infectious Disease Unit, Bambino Gesù Children’s Hospital, IRCCS, Rome, 00165 Italy; 5 Fondazione IRCCS Ca’Granda Ospedale Maggiore Policlinico, SC Pediatria Pneumoinfettivologia, Milan, Italy; 6 Pediatric Infectious Diseases Unit, Regina Margherita Children’s Hospital, University of Turin, Turin, Italy; 7 Department of Pediatrics and “A. Nocivelli” Institute for Molecular Medicine, Department of Clinical and Experimental Sciences, ASST Spedali Civili of Brescia, University of Brescia, Brescia, Italy; 8 Pediatric Infectious Disease Unit, Salesi Children Hospital, Azienda Ospedaliero Universitaria Delle Marche, Ancona, 60121 Italy; 9 Department of Medical and Surgical Sciences, Alma Mater Studiorum, University of Bologna, Bologna, Italy; 10 Pediatric Emergency Unit, IRCCS Azienda Ospedaliero-Universitaria Di Bologna, Bologna, Italy; 11 Department of Maternal, Infantile and Urological Sciences, Sapienza University of Rome, Rome, 00161 Italy; 12 Division of Pediatrics, University Hospital of Udine, P.Zzale S. Maria Della Misericordia, Udine, 15. 33100 Italy; 13 Department of Woman and Child Health and Public Health, Fondazione Policlinico Universitario A. Gemelli IRCCS, Rome, Italy; 14 Department of Health Promotion, Mother and Child Care, Internal Medicine and Medical Specialties“G D’Alessandro”, University of Palermo, Division of Paediatric Infectious Disease,“G. Di Cristina”Hospital, ARNAS Civico Di Cristina Benfratelli, PalermoPalermo, Italy; 15 University of Milan, Milan, Italy; 16 Pediatric Unit, San Martino Hospital, Belluno, 32100 Italy; 17 Institute of Child Health, Bambino Gesù Children’s Hospital, IRCCS, Rome, Italy; 18 Pediatric Infectious Disease Unit, Department of Translational Medical Sciences, University Hospital Policlinico Federico IIand, University of Naples Federico II, Naples, 80131 Italy

**Keywords:** Group A Streptococcal Infections, Children, Invasive disease, Streptococcus pyogenes

## Abstract

**Background:**

Group A *Streptococcus* causes pediatric infections from mild to severe forms. Since late 2022, invasive cases have increased in Europe, possibly due to reduced post-COVID-19 immunity, more respiratory virus circulation, and emergence of virulent strains.

**Methods:**

A retrospective, multicenter observational study was conducted in twelve Italian pediatric Hospitals, including patients under 18 years hospitalized with invasive or severe Group A *Streptococcus* infection. Data were anonymized and analyzed to identify factors associated with Pediatric Intensive Care Unit (PICU) admission and discharge with sequelae or death.

**Results:**

Seventy-five children with invasive or severe Group A Streptococcus infection were included; the majority (69.3%) were aged 2–10 years. Invasive Group A *Streptococcus* (iGAS) infection accounted for 58.7% (*n* = 44) and severe GAS (sGAS) infection for 41.3% (*n* = 31) of cases. Pediatric Intensive Care Unit admission was required in 45.3% (*n* = 34) of the entire patient cohort, in this subgroup viral coinfection (OR 5.684, *p* = 0.003), sepsis/septic shock (OR 4.406, *p* = 0.003), iGAS diagnosis (OR 4.153, *p* = 0.005), and procalcitonin (PCT) > 0.5 ng/mL (OR 7.105, *p* = 0.019) were independently associated with admission; the use of corticosteroids (OR 4.641, *p* = 0.003) and intravenous immunoglobulin (IVIG) (OR 16.667, *p* = 0.003) was also significantly more frequent.

All patients received empirical β-lactam antibiotics; anti-toxin therapy was administered in 47 patients (62.7%): clindamycin (49.3%), linezolid (16.0%), and rifampicin (1.3%). Mechanical ventilation was required in 24.0% (*n* = 18), and 49.3% (*n* = 37) underwent surgery. Post-infectious sequelae occurred in 20.0% (*n* = 15) and four children died, mostly due to streptococcal toxic shock syndrome.

**Conclusion:**

Pediatric invasive group A streptococcal infection continues to pose a significant clinical challenge, with notable rates of morbidity and mortality, underscoring the need for early recognition and close monitoring of high-risk patients. A widespread use of adjunctive therapies was documented. Continued surveillance and robust clinical research are essential to optimize management strategies and improve patient outcomes.

**Supplementary Information:**

The online version contains supplementary material available at 10.1186/s13052-025-02103-7.

## Background

Group A *Streptococcus* (GAS) is a gram-positive bacterium causing a range of diseases, from superficial infections to severe immune-mediated complications. Its virulence relies on mechanisms of tissue invasion and immune evasion, with distinct serotypes showing specific tissue tropism [1].

GAS is classified into over 220 *emm* (M) types, based on sequence variation in the gene encoding the M protein, a key surface virulence factor. These *emm* types show distinct geographic distribution patterns [2–4].

Since the 1980 s, the emm1.0 type has predominated among invasive GAS strains in industrialized countries [5]. The rising incidence of iGAS has been linked to the global spread of hypervirulent clones, notably M1global (M1T1) [6, 7]. In 2019 the M1UK variant emerged in the United Kingdom (UK) during a surge in scarlet fever and iGAS cases [8], distinguished from M1global by 27 single nucleotide polymorphisms (SNPs). M1UK exhibits enhanced *SpeA* production driven by a *ssrA* gene mutation [9]. This lineage has since been detected across multiple countries, including the United States of America (USA), Canada, the Netherlands, Australia, and Taiwan [10]. Recent genomic surveillance from the UK, Iceland, Denmark, and the Netherlands suggests that the spread of M1 variants, such as M1UK and M1DK, potentially associated with increased *SpeA* expression, may be contributing to the current rise in iGAS cases [11–14].

Historically, prior to the COVID-19 pandemic, iGAS incidence in Western countries was low, ranging between 1.5 and 3.8 cases per 100.000 per year, and was highest in children under one year [15–19]. After pandemic period in Italy, Mangioni et al. showed an increased incidence of iGAS infections especially in adult patients [20, 21]

Recent research by Mariani et al. explored the predictors of iGAS infection, linking factors such as varicella infection, chronic comorbidities, specific GAS virulence factors, Nonsteroidal anti-inflammatory drugs (NSAIDs) usage, socioeconomic status, and recent non-invasive GAS infections to an elevated risk of iGAS in children. [22]

Streptococcal toxic shock syndrome (STSS) is a serious complication of iGAS, marked by hypotension, organ failure, and rash. STSS carries a high mortality rate, exceeding 25% within 24 h in certain studies [23]. Recent scientific evidence, primarily from adult studies, indicates that adjunctive IVIG alongside clindamycin treatment in STSS patients leads to a statistically significant reduction in mortality [23–25].

This study aimed to investigate patients with iGAS or sGAS infections, admitted both in wards and in pediatric intensive care units in twelve Italian pediatric hospitals. We assessed the increase in case numbers compared to previous years and aimed to identify factors predisposing to the admission to the PICU. We also analyzed the factors associated with discharge with sequelae or correlated with mortality, with a particular focus on the role of antibiotic and corticosteroid use in the PICU and general wards, as well as the impact of antibiotics and non-steroid anti-inflammatory drugs (NSAIDs) used prior to diagnosis during home care.

## Material and methods

### Population and study design

We conducted an observational retrospective multicenter study, including data from children with iGAS or severe GAS infection admitted to 12 Italian hospitals (within the Italian Society of Pediatric Infectious Diseases network) from September 1, 2022, to August 31, 2023.

The inclusion criteria comprised individuals under 18 years of age necessitating hospitalization for iGAS or sGAS infections (according to the definitions below). Exclusion criteria included patients older than 18 years.

We conducted a retrospective review of hospitalization records and medical charts from all eligible participants. This process included the extraction of key patient information, such as medical history, demographic characteristics, and baseline clinical data. The variables collected are summarized as follows:

- Demographic and background data: gender, age, place of birth, parental nationality and place of birth, presence of comorbidities or immunodeficiencies, surgical procedures performed within 30 days prior to admission, use of antibiotics or NSAIDs within the preceding 15 days, and recent international travel.
- Clinical presentation at disease onset.
- Laboratory results at admission: white blood cell (WBC) count, absolute neutrophil count, C-reactive protein (CRP), procalcitonin, and urinalysis.
- Results of rapid streptococcal antigen detection tests (pharyngeal swabs).
- Microbiological findings: including blood cultures, blood polymerase chain reaction (PCR) assays, cultures and PCR from other biological specimens, and antibiotic susceptibility profiles.
- Evidence of co-infections (bacterial or viral).
- Radiological assessments: including ultrasound, X-ray, computerized tomography scan, Magnetic Resonance Imaging, or other imaging modalities.
- Treatment details: type, duration, dosage, and route of antibiotic administration; use of adjunctive therapies such as corticosteroids or intravenous immunoglobulin; and any therapy-related adverse events.
- Non-pharmacological interventions: including surgery, invasive ventilatory support, and extracorporeal techniques if applicable.
- Discharge outcomes: total duration of hospitalization, admission to the Pediatric Intensive Care Unit, final discharge diagnosis, and the presence of sequelae or mortality.

NSAID exposure was defined as the administration of at least one dose within the fifteen days preceding hospital admission. Owing to the limited sample size, stratification of patients based on NSAID dosage or duration of use was not feasible. Similarly, antibiotic exposure was defined as the receipt of at least one dose of any antibiotic within the month prior to admission. Given the small number of cases, classification by antibiotic class or agent was not feasible. Further stratification of either variable would have led to excessive fragmentation and compromised statistical power.

Each participating hospital collected data on patients enrolled in the study, which were subsequently entered into a centralized database and analyzed.

The geographical distribution of the participating centers was representative sample across the Italian territory, for more details see Supplemental material, Table S4.

**Fig. 1:**
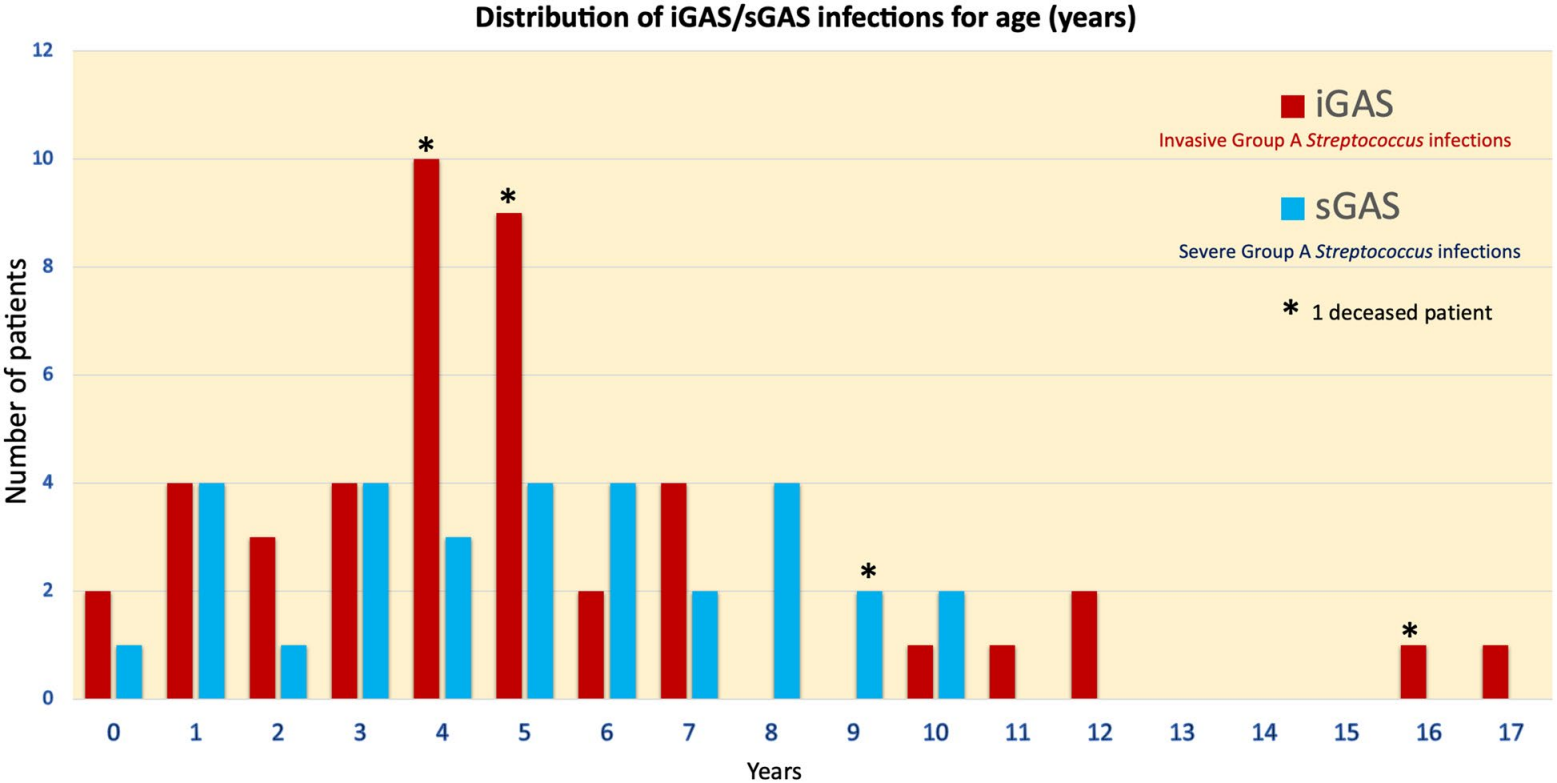
Distribution of iGAS/sGAS infections by age (per years)

**Fig. 2:**
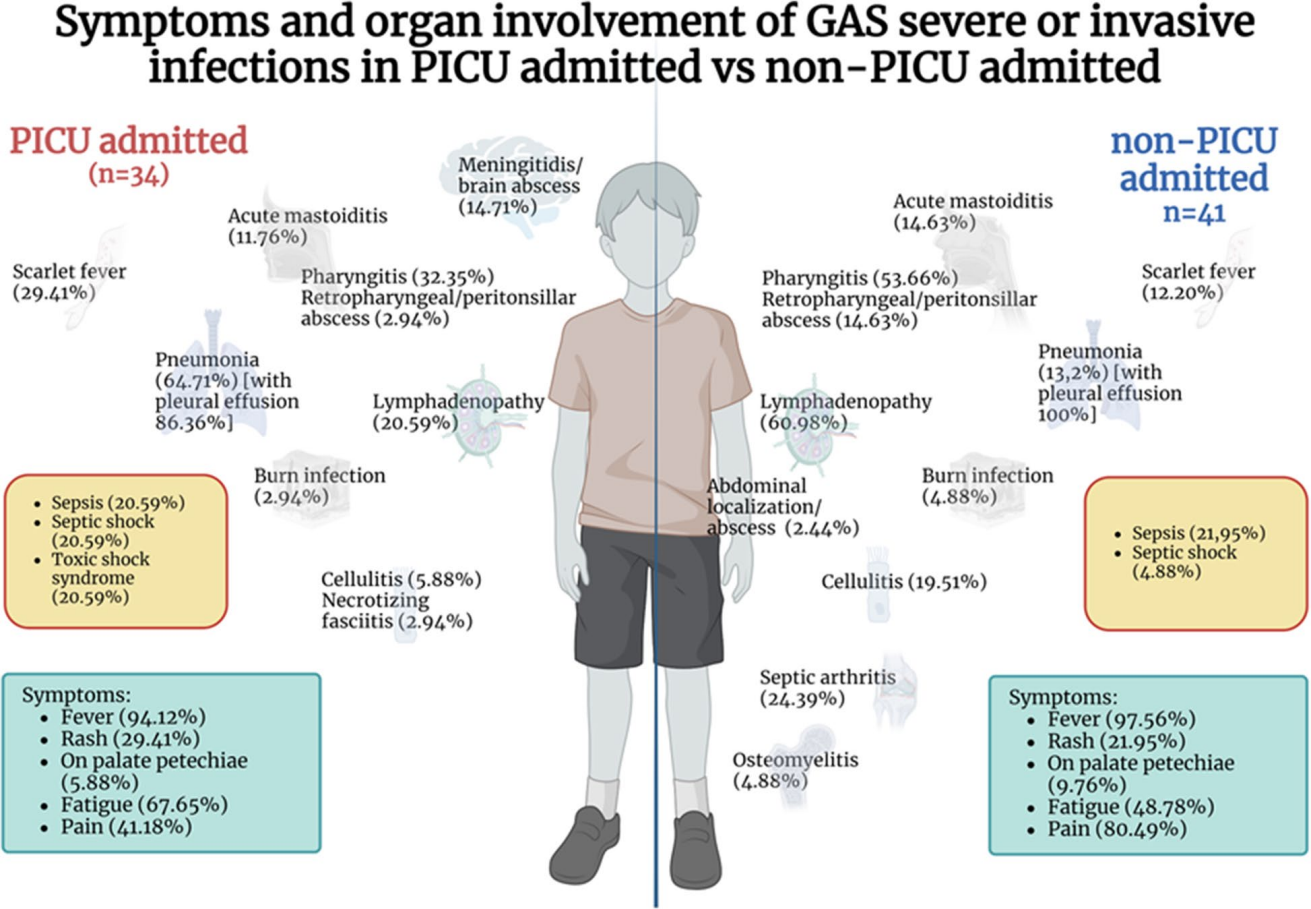
Clinical features and organ involvement in children admitted or not to Paediatric Intensive Care (PICU) Created with BioRender.com

The clinical characteristics of the patients included in the study were analyzed by stratifying the cohort into two groups: those who required admission to the pediatric intensive care unit and those managed in general wards (Table 1, Figs 1 and 2). Additionally, patients were categorized based on infection type, distinguishing those with invasive group A streptococcal disease from those with severe GAS infections (Supplemental material Table S3).

**Table 1:** Characteristics of the study children admitted or not to Paediatric Intensive Care Unit (PICU)

	**PICU patients** *(N* = *34)*	**Non-PICU patients** *(N* = *41)*	**Total** *(N* = *75)*	***p*-value**
**Age (months; median and IQR)**	60.00 [37.33–71.50]	64.00 [46.00–94.00]	61.00 [43.90–84.00]	0.4122
Age groups (n; %):				
- 0–2 years	7 (20.6%)	8 (19.5%)	15 (20.0%)	0.9077
- 3–10 years	24 (70.6%)	28 (68.3%)	52 (69.3%)	0.8301
- > 10 years	3 (8.8%)	5 (12.2%)	8 (10.7%)	0.7216
**Gender (male; ***n*; %)	19 (55.9%)	29 (70.7%)	48 (64.0%)	0.1823
**Type of GAS infection** (*n*; %)				
Invasive GAS infection	26 (76.5%)	18 (43.9%)	44 (58.7%)	**0.0044**
Severe GAS infection	8 (23.5%)	23 (56.1%)	31 (41.3%)	
**Comorbidities** (*n*; %)	8 (23.5%)	4 (9.8%)	12 (16.0%)	0.1249
**Italian region** (*n*; %):				
- Northern	12 (35.3%)	19 (46.3%)	31 (41.3%)	0.3334
- Central	21 (61.8%)	18 (43.9%)	39 (52.0%)	0.1232
- Southern	1 (2.9%)	4 (9.8%)	5 (6.7%)	0.3692
**Onset Symptoms** (*n*; %)				
- Fever (> 38 °C)	32 (94.12%)	40 (97.6%)	72 (96.0%)	0.5871
- Rash	10 (29.4%)	9 (21.9%)	19 (25.3%)	0.4596
- Petechiae on palate	2 (5.9%)	4 (9.8%)	6 (8.0%)	0.6832
- Fatigue	23 (67.7%)	20 (48.8%)	43 (57.3%)	0.1001
- Osteo-muscular pain	3 (8.8%)	13 (31.7%)	16 (21.3%)	**0.0254**
- Sore throat	2 (5.9%)	9 (21.9%)	11 (14.7%)	0.0980
- Otalgia (earache)	4 (11.8%)	4 (9.8%)	8 (10.7%)	1
- Abdominal pain	6 (17.7%)	2 (4.9%)	8 (10.7%)	0.1305
- Headache	1 (2.9%)	1 (2.4%)	2 (2.7%)	1
**Organ involvement** (*n*; %)				
- Pharyngitis - Acute mastoiditis - Retropharyngeal/peritonsillar abscess	11 (32.4%) 4 (11.8%) 1 (2.9%)	22 (53.7%) 6 (14.6%) 6 (14.6%)	33 (44.0%) 10 (13.3%) 7 (9.3%)	0.0643 0.7484 0.1188
- Lymphadenopathy - Pneumonia [with pleural effusion] - Abdominal abscess/localization - Meningitidis/cerebral abscess - Septic arthritis - Osteomyelitis - Cellulitis - Necrotizing fasciitis - Burn/wound infection	7 (20.6%) 22 (64.7%) [19 (86.4%)] 0 (0.0%) 5 (14.7%) 0 (0.0%) 0 (0.0%) 2 (5.9%) 1 (2.9%) 1 (2.9%)	25 (60.9%) 6 (14.6%) [6 (100.0%)] 1 (2.4%) 0 (0.0%) 10 (24.4%) 2 (4.9%) 8 (19.5%) 0 (0.0%) 2 (4.9%)	32 (42.7%) 28 (37.3%) [25 (89.3%)] 1 (1.3%) 5 (6.7%) 10 (13.3%) 2 (2.7%) 10 (13.3%) 1 (1.3%) 3 (4.0%)	**0.0004** ** < 0.0001** 1 **0.0161** **0.0015** 0.4977 0.1011 0.4533 1
**Sepsis** (*n*; %) **Septic shock** (*n*; %) **Toxic shock syndrome** (*n*; %)	7 (20.6%) 7 (20.6%) 7 (20.6%)	9 (21.9%) 2 (4.9%) 0 (0.0%)	16 (21.3%) 9 (12.0%) 7 (9.3%)	0.8859 0.0701 **0.0027**
**C-reactive protein (CRP) at onset**				
CRP serum levels (mg/dl; median and IQR)	19.69 [13.35–27.22]	11.30 [6.40–18.46]	15.77 [8.22–23.85]	**0.0074**
- Groups (mg/dl; *n*; %): 1. ≤ 15.9 2. 16.0–29.9 3. ≥ 30.0	12 (35.3%) 15 (44.1%) 7 (20.6%)	27 (65.9%) 10 (24.4%) 4 (9.8%)	39 (52.0%) 25 (33.3%) 11 (14.7%)	**0.0084** 0.0712 0.2092
**Procalcitonin (PCT) serum levels (ng/ml) at onset** (*N* = *60*)	(*N* = 29)	(*N* = 31)		
- PCT serum levels (mg/dl; median and IQR) - Negative (≤ 0.5) - Positive (> 0.5)	28.50 [4.26–101.00] 1 (3.5%) 28 (96.6%)	1.20 [0.25–12.63] 10 (32.3%) 21 (67.7%)	12.40 [0.92–34.75] 11 (18.3%) 49 (81.7%)	** < 0.0001** **0.0059**
**White blood cells (WBC)** *(N* = *74)*	(*N* = 33)	(*N* = 41)		
- WBC count (cell/mm^3^; median and IQR) - Groups (cell/mm^3^; n; %):	12,970.00 [7540.00–18370.00]	12,810.00 [8910.00–21690.00]	12,890.00 [8715.00–19832.50]	0.2757
1. < 4000 2. 4000–20000 3. > 20,000	5 (15.2%) 23 (69.7%) 5 (15.2%)	1 (2.4%) 27 (65.9%) 13 (31.7%)	6 (8.1%) 50 (67.6%) 18 (24.3%)	0.0828 0.7255 0.1121
**Neutrophils** *(N* = *72)*	(*N* = 32)	(*N* = 40)		
- Neutrophils count (cell/mm^3^; median and IQR)	8827.00 [3962.00–15672.00]	8928.00 [5457.50–18367.50]	8928.00 [4457.00–16542.75]	0.4533
- Groups (cell/mm^3^; *n*; %): 2. ≤ 10,000 3. > 10,000	18 (56.3%) 14 (43.8%)	21 (52.5%) 19 (47.5%)	39 (54.2%) 33 (45.8%)	0.7510
**Microbiological findings** (*n*; %)				
- Positive blood culture - Positive blood PCR - Positive culture on other sites - Positive PCR on other sites - Positive rapid antigenic pharyngeal test (*N* = 28)	18 (52.9%) 2 (5.9%) 7 (20.6%) 2 (5.9%) 9 (81.8%) (*N* = 11)	4 (9.8%) 5 (12.2%) 16 (39.0%) 9 (21.9%) 16 (94.1%) (*N* = 17)	22 (29.3%) 7 (9.3%) 23 (30.7%) 11 (14.7%) 25 (89.3%)	**0.0001** 0.4456 0.0848 0.0980 0.5433
**Respiratory coinfections** (*n*; %)				
Bacterial coinfections	5 (14.7%)	7 (17.1%)	12 (16.0%)	1
Viral coinfections	9 (26.5%)	10 (24.4%)	19 (25.3%)	
**Antibiotics therapy duration (days; median and IQR)**				
- Intravenous therapy	16.00 [11.25–28.00]	14.00 [9.00–21.00]	15.00 [9.50–22.00]	0.1902
- Oral therapy *(N* = *42)*	10.00 [8.00–14.00] (*N* = 19)	13.00 [7.25- 19.25] (*N* = 23)	11.00 [8.00–18.50]	0.8103
**Use of anti-toxin antibiotics** (*n*; %)				
- Clindamycin - Linezolid - Clindamycin OR Linezolid	18 (52.9%) 7 (20.6%) 23 (67.7%)	19 (46.3%) 5 (12.2%) 23 (56.1%)	37 (49.3%) 12 (16.0%) 46 (61.3%)	0.5693 0.3594 0.3066
**Time between diagnosis and anti-toxin antibiotics start** (days; median and IQR)	1.00 [0.00–5.00]	1.00 [0.00–2.00]	1.00 [0.00–3.00]	0.8259
**Antibiotic resistant GAS (n; %)** *(N* = *40)*	(*N* = 24) 4 (16.7%)	(*N* = 16) 2 (12.5%)	6 (15.0%)	0.4011
**Hospitalization duration (days; median and IQR)** **Surgery** (*n*; %) **Glucocorticoids** (*n*; %) **Need of IVIG** (*n*; %) **Need of invasive ventilation** (*n*; %)	20.00 [11.25–29.75] 12 (35.3%) 12 (35.3%) 9 (26.5%) 15 (44.1%)	13.00 [9.00–21.00] 25 (60.9%) 14 (34.2%) 2 (4.9%) 3 (7.3%)	16.00 [9.00–25.00] 37 (49.3%) 26 (34.7%) 11 (14.7%) 18 (24.0%)	**0.0264** **0.0268** 0.9172 **0.0184** **0.0003**
**Discharged without sequalae** (*n*; %) **Sequelae** (*n*; %) **Death** (*n*; %)	23 (67.7%) 6 (17.7%) 3 (8.8%)	33 (80.5%) 9 (21.9%) 1 (2.4%)	56 (74.7%) 15 (20.0%) 4 (5.3%)	0.2031 0.6427 0.3233

*GAS* Group A β-Haemolytic Streptococcus, *IQR* Interquartile range, *IVIG* intravenous immunoglobulins, *PICU* Paediatric Intensive Care Unit, *PCR* Polymerase Chain Reaction

^*^ 12 children presented co-morbidities including 3 children with acquired immunodeficiency, respectively one for a previous T-cell acute lymphoblastic leukemia, and 2 patients for an iatrogenic immunodeficiency due to therapy for Juvenile Idiopathic Arthritis, and Ulcerative Colitis

A comprehensive analytical strategy was adopted, incorporating both univariate and multivariate logistic regression analyses to identify potential risk factors associated with PICU admission and with adverse outcomes, including discharge with sequelae or death, across the entire cohort (Table 3 and Supplemental material Table S2). Univariate analyses were also conducted to explore differences in clinical presentation, laboratory findings, and outcomes between iGAS and severe GAS cases (Supplemental material Table S4).

Given the multicentric and observational nature of the study, diagnostic evaluations—including laboratory, microbiological, and radiological investigations—were performed in accordance with local clinical practices and physician judgment, ensuring individualized patient care and optimized management strategies.

### Definitions

Definitions of invasive Group A Streptococcus and severe GAS infection applied in this study were derived from the most recent guidance issued by the UK Health Security Agency [26].

Invasive GAS infection was defined by the identification of *S. pyogenes* through culture or validated molecular techniques from a sterile anatomical site, including blood, cerebrospinal fluid, joint aspirate, pericardial-peritoneal-pleural fluids, bone, deep tissue, or deep abscess during surgical intervention or post-mortem examination [26–30]

Severe GAS infection was defined by the identification of *S*. *pyogenes* from a non-sterile site, such as the throat, sputum or wound, in conjunction with a severe clinical presentation, including streptococcal toxic shock syndrome, necrotizing fasciitis, pneumonia, septic arthritis, meningitis, peritonitis, osteomyelitis and myositis [26]

### Laboratory methods

Diagnostic evaluation of invasive or severe Group A *Streptococcus* infection and bacterial coinfections was conducted using conventional culture or molecular testing. Specimens for both diagnostic purposes were collected from blood, cerebrospinal fluid, joint aspirates, pericardial or pleural fluids, bone, deep tissue, or abscesses obtained during surgical procedures.

These diagnostic methodologies are well established and validated in the scientific literature [31]. Viral coinfections were detected through PCR analysis of blood, nasopharyngeal swabs, or other relevant biological samples. All microbiological analyses were performed in certified laboratories within tertiary or secondary-level healthcare institutions [31–36]

### Statistical analysis

Categorical data were compared using the Chi-squared test, or Fisher’s exact test when expected cell sizes were smaller than five. The Wilcoxon-Mann–Whitney test was used for continuous measurements in unpaired analysis, assuming the dependent variable was not normally distributed. Logistic regression models were employed to calculate odds ratios (ORs) and their corresponding 95% confidence intervals (CIs).

Outcomes were classified into three primary clinical endpoints to investigate factors associated with disease severity and prognosis. First, the study assessed predictors of PICU admission, considered a clinical indicator of acute disease severity, within the entire cohort of patients, including both iGAS and sGAS. Secondly, factors associated with a composite endpoint of in-hospital mortality and post-discharge sequelae were evaluated across the full cohort. Lastly, the analysis explored predictors of severe GAS disease presentation as compared to invasive GAS infections within the overall patient population.

The primary outcome variables were:

1. Admission to the Pediatric Intensive Care Unit
2. A composite outcome of in-hospital mortality and post-discharge sequelae
3. Occurrence of severe Group A *Streptococcus* infection

Univariate logistic regression analyses were initially performed to identify independent variables associated with key clinical outcomes. Variables included in the univariate models were type of infection (severe GAS vs. iGAS), site of infection, gender, and age at diagnosis (categorized as 0–2 years, 3–10 years, > 10 years). Additional demographic and clinical variables were place of birth (Italy vs. abroad), presence of comorbidities (absent or present, further categorized as neurological, respiratory, or other), receipt of antibiotics for any reason in the previous month, and use of NSAIDs within the 15 days prior to admission.

Clinical features at presentation were also analyzed, including the presence of fever, rash, petechiae on the palate, and various pain types (abdominal, osteoarticular, cervical/headache, sore throat, earache).

Among the complications observed during hospitalization were upper respiratory tract infections—including acute mastoiditis, retropharyngeal abscesses, lymphadenopathies, and lymphadenitis—as well as osteomyelitis or septic arthritis, meningitis, pharyngitis, skin infections, sepsis, and septic shock.

Timing of pediatric intensive care unit admission was categorized as occurring on day 1, after admission, or not required. Laboratory parameters at admission were stratified as follows: white blood cell count (< 4000/µL, 4000–20000/µL, > 20,000/µL), absolute neutrophil count (≤ 10,000/µL, > 10,000/µL), C-reactive protein levels (≤ 15.9 mg/dL, 16.0–29.9 mg/dL, ≥ 30.0 mg/dL), and procalcitonin (PCT) levels (≤ 0.5 ng/mL, > 0.5 ng/mL, or missing).

Microbiological variables included: detection of GAS in blood via culture or PCR (positive, negative, missing); GAS detection from non-blood samples (positive, negative, missing); respiratory viral co-infections identified via nasopharyngeal or throat swab PCR; and documented bacterial co-infections, including *Staphylococcus aureus*, as identified by culture or molecular methods.

Additional variables assessed were the need for surgical intervention, administration of intravenous immunoglobulins, use of corticosteroids, and details of antibiotic therapy (type, weight-adjusted dosage, duration, mode of administration). Timing of anti-toxin antibiotic initiation relative to hospital admission was also included (< 2 days vs. ≥ 2 days).

Variables demonstrating statistical significance in univariate analysis (p < 0.05) were subsequently entered into multivariate logistic regression models to identify independent predictors of outcomes.

All analyses were performed using StataCorp. 2023. Stata Statistical Software: Release 18. College Station, TX: StataCorp LLC.

## Results

A total of 75 children with invasive or severe Group A *Streptococcus* infection were included. The cohort demonstrated a balanced geographic distribution across Italy: 28 patients (37.3%) were enrolled from Northern Italy (Milan, Turin, Udine, Brescia), 25 (33.3%) from Central Italy (Florence, Ancona, Bologna), and 22 (29.3%) from Southern Italy (Naples, Rome, Palermo). Most children (69.3%) were aged 2–10 years. Of the 75 cases, 44 (58.7%) met criteria for invasive GAS infection, while 31 (41.3%) were classified as severe GAS infection (Fig. 1).

### Clinical characteristics

Among patients with iGAS, 40% required admission to the pediatric intensive care unit. Fever (97.7%) and localized pain, particularly abdominal or joint pain, were the most prevalent symptoms. Complications included mastoiditis or retropharyngeal abscesses in 34.0%, and systemic involvement (e.g., sepsis or septic shock) in 56.8% (Fig. 2).

In the sGAS group, 25% required PICU admission. Fever (93.5%) and pharyngitis (64.5%) were common presenting features. During Hospitalisation, 67.7% developed complications, including otomastoiditis, retropharyngeal abscesses, or lymphadenopathy (Fig. 2).

The univariate analysis did not identify any possible risk factors predisposing to iGAS with respect to sGAS. For more details see Supplemental Material Table S3.

### Microbiology and antibiotic susceptibility

Blood cultures were positive in 53.7% (21/41) of iGAS cases and blood PCR testing was positive in 65.2% (15/23). Among sGAS patients, culture positivity from peritonsillar drainage was 83.3% (5/6), the PCR confirming infection in the only sample in which it was performed (Table 2).

**Table 2:** Sites of isolation of *S. pyogenes* divided into the two subgroups iGAS (invasive GAS infections) and severe GAS infections. GAS: Group A β-Hemolytic Streptococcus; PCR: Polymerase Chain Reaction

	**Site of GAS isolation**	**Culture (n/N; %)**	**Polymerase chain reaction (PCR) (n/N; %)**
**iGAS** (*N* = 44)	Blood	22/41 (53.7%)	15/23 (65.2%)
	Pleural fluid	10/11 (90.9%)	6/6 (100.0%)
	Liquor	2/3 (66.7%)	0/0 (0.0%)
	Synovial fluid	3/4 (75.0%)	2/2 (100.0%)
	Broncho-aspiration	1/1 (100.0%)	0/0 (0.0%)
	Subperiosteal abscess	0/1 (0.0%)	1/1 (100.0%)
**Severe GAS infections** (*N* = 31)	Peritonsillar abscess drainage	5/6 (83.3%)	1/1 (100.0%)
	Auricular drainage	3/4 (75.0%)	2/2 (100.0%)
	Pharyngeal swab	1/2 (50.0%)	1/1 (100.0%)
	Wound/cutaneous swab	1/1 (100.0%)	1/1 (100.0%)
	Bronchoalveolar Lavage	1/3 (33.3%)	2/2 (100.0%)
	Lymphadenitis drainage/biopsy	0/2 (0.0%)	2/2 (100.0%)

Antibiotic susceptibility testing was performed in 40 patients (53.3%) of the overall population. Resistance was identified in 6 cases (15.0%), five of the six resistant *Streptococcus pyogenes* strains demonstrated resistance to more than one antibiotic: erythromycin (6 strains, 15.0%), tetracycline (4 strains, 10.0%), and trimethoprim/sulfamethoxazole (2 strains, 5.0%). One isolate (2.5%) showed resistance to each of azithromycin, clindamycin, levofloxacin, rifampicin, and minocycline. Five of six resistant strains demonstrated multi-drug resistance.

### Coinfections

Viral coinfections were identified in 25.3% (*n* = 19) and bacterial coinfections in 16.0% (*n* = 12) of the total cases included in this study. Among iGAS cases, viral coinfections occurred in 31.8%, most commonly involving Rhinovirus and Adenovirus. For more details see Supplemental material, Table S1.

Viral coinfections require only supportive care, while bacterial coinfections require antibiotic therapy based on susceptibility test [36].

### Treatment and supportive care

Median Hospital stay was 16.0 days (IQR 9.0–25.0). Intravenous antibiotic therapy had a median duration of 15.0 days (IQR 9.5–22.0), and oral therapy 11.0 days (IQR 8.0–18.5). All patients received empirical β-lactam antibiotics. Anti-toxin therapy was administered in 47 patients (62.7%): clindamycin (49.3%), linezolid (16.0%), and rifampicin (1.3%) (Table 1).

Systemic glucocorticoids were used in 34.7% and IVIG in 14.7% (9/11 of these were PICU patients). Inotropic support was needed in 10.7%. Two children (2.7%) underwent continuous venovenous hemofiltration; 18/75 (24.0%) required invasive mechanical ventilation. No patient received extracorporeal membrane oxygenation.

### Surgical interventions and outcomes

Surgical procedures were performed in 49.3% of cases. Thoracocentesis (22.7%) and abscess drainage (17.3%) were most common. Other interventions included arthrocentesis (5.3%), neurosurgery (2.7%), and fasciotomy (1.3%).

Post-infectious sequelae occurred in 15 children (20.0%), more frequently among PICU patients (29.4% vs 12.2%). Sequelae included respiratory dysfunction, neurological deficits, and musculoskeletal complications.

Four children (5.3%) died. Three deaths were due to streptococcal toxic shock syndrome. All presented with fever; blood cultures were positive in one patient, while PCR for GAS on cerebrospinal, pleural, or bronchoalveolar lavage fluid was positive in the other three. All these patients were admitted to PICU and required mechanical ventilation. Two of them received anti-toxin antibiotics and three were treated glucocorticoids and IVIG.

### Risk factors for PICU admission

Univariate analysis identified several factors associated with PICU admission: sepsis or septic shock (OR 4.41; *p* = 0.003), PCT > 0.5 ng/mL (OR 7.11; *p* = 0.019), viral respiratory coinfection (OR 5.68; *p* = 0.003), IVIG use (OR 16.67; *p* = 0.009), glucocorticoid therapy (OR 4.64; *p* = 0.003), death or sequelae (OR 5.04; *p* = 0.006), and diagnosis of iGAS (OR 4.15; *p* = 0.005). Multivariate analysis confirmed two independent risk factors: viral coinfection (OR 4.80; 95% CI 1.09–21.27; *p* = 0.039) and systemic glucocorticoid use (OR 5.86; 95% CI 1.47–23.38; *p* = 0.012). (Table 3, Fig. 2).

**Table 3:** Univariate analysis for factors associated with Paediatric Intensive Care Unit (PICU) admission

	**Univariate analysis**			
**Study population characteristics**	**n/N**	**OR**	**95% CI**	***p***
Male Female	19/48 (39.6%) 15/27 (55.6%)	1 1.908	0.735–4.955	0.185
Age - 0–2 - 3–10 - > 10	7/15 (46.7%) 24/52 (46.2%) 3/8 (37.5%)	1 0.980 0.686	0.310–3.099 0.119–3.963	0.972 0.673
Birthplace - Italy - Another country	29/66 (43.9%) 5/9 (55.6%)	1 1.595	0.393–6.479	0.514
Comorbidities - No - Yes Type of comorbidities - Others - Neurologic - No - Respiratory	26/62 (41.9%) 8/13 (61.5%) 2/3 (66.7%) 5/6 (83.3%) 26/63 (41.3%) 1/3 (33.3%)	1 2.846 1 2.500 0.351 0.250	0.775–10.452 0.100–62.605 0.030–4.081 0.008–7.452	0.115 0.577 0.403 0.423
Antibiotics in the previous month - No - Yes - Missing	22/50 (44.0%) 7/19 (36.8%) 5/6 (83.3%)	1 0.742 6.364	0.250–2.201 0.692–58.502	0.591 0.102
NSAIDs in the previous 15 days - No - Yes - Missing	15/41 (36.6%) 13/26 (50.0%) 6/8 (75.0%)	1 1.733 5.200	0.639–4.700 0.929–29.094	0.280 0.061
Fever - No - Yes	2/3 (66.7%) 32/72 (44.4%)	1 0.4	0.035–4.612	0.463
Rash - No - Yes	24/56 (42.9%) 10/19 (52.6%)	1 1.481	0.521–4.211	0.461
On palate petechiae - No - Yes	32/69 (46.4%) 2/6 (33.3%)	1 0.578	0.099–3.367	0.542
Pain - No - Yes Types of pain - Abdominal - Other - Osteo-articular - Cervical pain/headache - Pharyngeal - No - Earache	20/28 (71.4%) 14/47 (29.8%) 6/8 (75.0%) 1/3 (33.3%) 2/16 (12.5%) 1/4 (25.0%) 2/10 (20.0%) 20/28 (71.4%) 2/6 (33.3%)	1 0.170 1 0.167 0.048 0.111 0.083 0.833 0.167	0.061–0.476 0.009–2.984 0.005–0.422 0.007–1.776 0.008–0.773 0.138–5.032 0.016–1.718	**0.001** 0.224 **0.006** 0.120 **0.029** 0.842 0.132
Upper airways infections* - No - Yes	24/39 (61.5%) 10/36 (27.8%)	1 0.240	0.091–0.637	**0.004**
Osteomyelitis and Septic Arthritis - No - Yes	33/62 (53.2%) 1/13 (7.7%)	1 0.073	0.009–0.598	**0.015**
Meningitidis - No - Yes	29/68 (42.7%) 5/7 (71.4%)	1 0.413	0.609–18.566	0.164
Pharyngitis - No - Yes	23/42 (54.8%) 11/33 (33.3%)	1 0.413	0.161–1.063	0.067
Cutaneous localization - No - Yes	32/65 (49.2%) 2/10 (20.0%)	1 0.258	0.051–1.308	0.102
Sepsis and septic shock - No - Yes	13/43 (30.2%) 21/32 (65.6%)	1 4.406	1.658–11.710	**0.003**
White Blood Count: - < 4000/mm^3^ - 4000–20000/mm^3^ - > 20,000/mm^3^ WBC ≥ 20,000/mm^3^ - No - Yes WBC < 4000/mm^3^ - No - Yes	5/6 (83.3%) 24/51 (47.1%) 5/18 (27.8%) 29/57 (50.9%) 5/18 (27.8%) 29/69 (42.0%) 5/6 (83.3%)	1 0.178 0.077 1 0.371 1 6.897	0.019–1.631 0.007–0.833 0.117–1.178 0.764–62.217	**0.127** **0.035** 0.093 0.085
Neutrophils - ≤ 10,000/mm^3^ - > 10,000/mm^3^	17/39 (43.6%) 17/36 (47.2%)	1 1.158	0.466–2.878	0.752
C-reactive protein (CRP) - CRP ≤ 15.9 mg/dl - CRP 16.0–29.9 mg/dl - CRP ≥ 30.0 mg/dl	12/37 (32.4%) 15/27 (55.6%) 7/11 (63.6%)	1 2.604 3.646	0.935–7.256 0.892–14.906	0.067 0.072
Positive Procalcitonin - PCT ≤ 0.5 ng/dl - PCT > 0.5 ng/dl - Missing	2/11 (18.2%) 30/49 (61.2%) 2/15 (13.3%)	1 7.105 0.692	1.383–36.497 0.082–5.863	**0.019** 0.736
Positive GAS blood culture or polymerase chain reaction (PCR) - No - Yes	18/48 (37.5%) 16/27 (59.3%)	1 2.424	0.924–6.362	0.072
Positive GAS culture or PCR on other sites - No - Yes - Missing	18/33 (54.6%) 13/26 (50.0%) 3/16 (18.8%)	1 0.833 0.192	0.298–2.334 0.046–0.804	0.729 0.024
Viral coinfections - No - Yes	19/55 (34.6%) 15/20 (75.0%)	1 5.684	1.791–18.036	**0.003**
Bacterial coinfections - No - Yes - *S. aureus* infections	22/57 (38.6%) 8/12 (66.7%) 4/6 (66.7%)	1 3.182 3.182	0.856–11.832 0.537–18.852	0.084 0.202
Surgery - No - Surgery - Drainage	17/38 (44.7%) 4/10 (40.0%) 13/27 (48.2%)	1 0.824 1.147	0.200–3.399 0.427–3.085	0.788 0.786
Intravenous immunoglobulin (IVIG) use: - No - Yes	24/64 (37.5%) 10/11 (90.9%)	1 16.667	2.006–138.437	**0.009**
Steroids during hospitalization - No - Yes	16/49 (32.7%) 18/26 (69.2%)	1 4.641	1.666–12.931	**0.003**
Time between diagnosis and anti-toxin antibiotics start - In the first 2 days - Over 2 days (≥ 2 days)	17/33 (51.5%) 17/42 (40.5%)	1 0.640	0.255–1.605	0.342
Outcome - Discharged without sequalae - Sequelae (death, sequelae)	20/56 (35.7%) 14/19 (73.7%)	1 5.040	1.583–16.049	**0.006**
Severe GAS infections iGAS	8/31 (25.8%) 26/44 (59.1%)	1 4.153	1.521–11.336	**0.005**

^*^In Upper airways infections are included: acute mastoiditis, retropharyngeal abscesses, lymphadenopathies, and lymphadenitis

### Risk factors for death or sequelae

Univariate analysis identified significant associations with iGAS diagnosis (OR 5.33; *p* = 0.014), NSAID (i.e., ibuprofen) use within 15 days pre-admission (OR 3.56; *p* = 0.027), ward-to-PICU transfer (OR 4.20; *p* = 0.053), and PICU admission (OR 5.04; *p* = 0.006).

On multivariate analysis, NSAID use (OR 4.02; 95% CI 1.04–15.48; *p* = 0.043) and ward-to-PICU transfer (OR 10.91; 95% CI 2.30–51.81; *p* = 0.003) remained significantly associated with adverse outcomes.

## Discussion

The COVID-19 pandemic precipitated the global adoption of unprecedented non-pharmaceutical interventions—including lockdowns, physical distancing, and universal masking—which effectively limited the transmission of SARS-CoV-2 and other respiratory pathogens. However, in the aftermath of these measures, several European countries, notably the Netherlands, the United Kingdom, and Italy, have reported a significant resurgence of infectious diseases, disproportionately affecting the pediatric population [26, 37–39].

Since the latter months of 2022, at least six European countries, including France, Ireland, the Netherlands, the United Kingdom, Sweden, Norway, Denmark, and Germany, have reported a marked increase in iGAS infections [40–48]. Similar trends were also observed in Spain [49], although these findings were not subsequently corroborated [40]. Data from the United States in 2022 indicate that the incidence of iGAS cases among children rose earlier than usual in the season, between September and November.

Several hypotheses have been posited to explain this resurgence: the re-circulation of respiratory viruses, such as respiratory syncytial virus (RSV) and influenza—both of which were suppressed during the pandemic—may have facilitated bacterial superinfections [48–56]

Furthermore, the concept of "immunity debt", which refers to a reduction in population-level immune priming due to limited exposure to pathogens during the pandemic, has been suggested as a potential contributing factor [57].

Rümke et al. (UK) conclude that the unique circumstances surrounding a population with potentially lower pathogen-specific immunity, coupled with high incidences of predisposing viral infections, combined with the emergence of a more virulent and fit M1UK lineage, have created optimal conditions for the increase in iGAS and severe GAS [57].

Among our cohort, the case fatality rate (CFR) was 5.3% (95% CI: 1.47–13.10), consistent with prior reports from Ireland (5.9%), Denmark (4%), and the United Kingdom (6.7%) [20, 44, 58, 59]

In contrast, lower CFRs have been reported in Spain (1.8%) and Canada (1.0%). [12–14, 49–53, 60, 61]. In our study among patients with iGAS, the CFR was higher at 6.8% (95% CI: 1.43–18.66), highlighting the severe nature of this subgroup. More recently, in 2022, in high-income countries, including Australia, iGAS CFR was 1% (95%CI: 0.2–3.3; 3 deaths out of 84 cases) [61]. In a UK report, iGAS CFR was 8.4% (30 death in 2022), stable with respect to the previous year [42].

In multivariate analysis, prior use of NSAIDs was independently associated with death or discharge with sequelae, however, this result should be interpreted with caution. Indeed, in another analysis, no statistically significant association was observed between previous NSAID exposure and PICU admission in either univariate or multivariate models. Therefore, the association between NSAID use and risk of death or sequelae observed may be influenced by residual confounding or other sources of bias that should be considered.

Several previous studies have suggested that NSAIDs may impair the host inflammatory response, potentially exacerbating disease progression. A population-based case–control study reported a significant association between NSAID use in the two weeks preceding illness and iGAS infection (OR 10.64; *p* = 0.05) [62, 63]. Similarly, Lesko et al. reported an increased risk of iGAS in children with varicella who were treated with ibuprofen (OR 3.9; 95% CI: 1.3–12) [64]. While these findings are not definitive, they underscore the need for caution in the use of NSAIDs during the prodromal phase of infection and highlight the importance of further research.

Our data confirm that respiratory viral coinfections were significantly associated with PICU admission, both in univariate and multivariate analyses. This finding is aligned with previous retrospective studies, including Abo et al., who reported a relative risk of 1.9 (95% CI: 1.2–3) for severe outcomes in children with viral coinfections. [61–65]. Similarly, Lees et al. found a 66% prevalence of viral coinfections—primarily RSV—among children with GAS pneumonia and parapneumonic effusion [66]. These observations support the hypothesis that viral coinfections contribute to disease severity in iGAS [61].

In addition to viral coinfections, our data confirm that several factors were independently associated with PICU admission, including septic shock, elevated procalcitonin (PCT > 0.5 ng/mL), IVIG administration, systemic glucocorticoid use, and iGAS diagnosis. Elevated PCT and CRP at admission were strong predictors of disease severity, consistent with previous European studies [67–69]

We observed widespread use of adjunctive therapies such as IVIG and protein synthesis-inhibiting antibiotics (e.g., clindamycin, linezolid, rifampin). In vitro and animal studies suggest that IVIG may neutralize superantigens, enhance opsonization, and modulate systemic inflammation [24]. Adult studies suggest benefit from combining IVIG with clindamycin, though pediatric evidence remains limited [70–73]. An Australian prospective study reported a non-significant trend toward reduced mortality with adjunctive clindamycin (OR 0.31; 95% CI: 0.09–1.12), while another study linked clindamycin use to reduced readmission risk [18]

In our cohort of patient use of systemic steroids was associated with increased risk of sepsis, septic shock, and PICU admission, potentially reflecting confounding by indication. Although this association persisted in multivariate models, it may represent a marker of disease severity rather than a causal relationship. Prospective studies are needed to clarify the role of corticosteroids in this setting.

## Limitations

This study has several limitations. Its retrospective design may be subject to data omissions and reporting bias. Microbiological characterization of GAS strains was not performed, precluding analysis of strain-specific virulence. The study was conducted over a relatively short timeframe (12 months) and lacked standardized clinical management protocols, as treatment was based on clinician judgment at each center.

## Conclusions

Our multicentric retrospective study provides relevant data regarding mortality rate and risk factors associated with poor outcomes in pediatric invasive group A streptococcal infections. The observed case fatality rate aligns with international data and underscores the clinical severity of iGAS, particularly in patients requiring intensive care.

Respiratory viral coinfections and elevated inflammatory marker (PCT) at admission were significantly associated with more severe disease and increased risk of PICU admission.

We documented widespread use of adjunctive therapies, including clindamycin, linezolid, IVIG, and systemic corticosteroids. While these interventions are commonly employed in invasive and severe GAS cases in adults, their efficacy in pediatric settings remains to be fully established. Further controlled studies are needed to better define their role, particularly in the management of life-threatening iGAS.

Continued epidemiological surveillance and high-quality clinical research are essential to refine treatment approaches and improve outcomes in affected children.

## Supplementary Information

Supplementary material 1

Supplementary material 2

Supplementary material 3

Supplementary material 4

## Data Availability

The datasets generated and analyzed during the current study are available from the corresponding author upon reasonable request.
