# Supplementary material for: Invasive Group A Streptococcus infections in children during the post-pandemic period: results from a multicenter study in Italy
Source: Ital J Pediatr. 2025 Nov 28;51:312. doi: 10.1186/s13052-025-02103-7 (PMC12661756; doi:10.1186/s13052-025-02103-7)
Supplement: Supplementary file 1 — Supplementary material 1 [file 13052_2025_2103_MOESM1_ESM.docx]

**Table S1:** Viral and bacterial respiratory coinfections into the two subgroups iGAS and Severe GAS infections.

|  | Viral respiratory coinfections  (n=19; 47.4%) | Bacterial respiratory coinfections  (N=12; 16.0%) |
| --- | --- | --- |
| Invasive GAS  (N=44) | N=14/44 (31.8%)  (8 patients with more than a virus isolated)  - Rhinovirus (n=6/44)  - Adenovirus (n=5/44)  - Coronavirus (n=4/44)  - Parainfluenza virus (n=3/44)  - Metapneumovirus (n=3/44)  - Bocavirus (n=1/31)  - A H3N2 Influenza virus (n=1/31)  - Influenza virus (n=1/44) | N=5/44 (11.4%)  (1 patient with more than a bacterium isolated)   - Streptococcus pneumoniae (n=2/44) - Pseudomonas aeruginosa (n=1/44) - Acinetobacter parvus (n=1/44) - Moraxella catarrhalis (n=1/44) - Haemophilus influenzae (n=1/44) |
| Severe GAS infections  (N=31) | N=5/31 (16.1%)  (1 patient with more than a virus isolated)   - Metapneumovirus (n= 3/31) - Bocavirus (n=1/31) - Parainfluenza virus (n=1/31) - Coronavirus (n=1/31) | N=7/31 (22.6%)  (2 patients with more than a bacterium isolated)   - Moraxella catarrhalis (n=1/31) - Haemophilus influenzae (n=1/31) - Klebsiella pneumoniae (n=1/31) - Fusobacterium necrophorum (n=1/31) - Staphylococcus aureus (n=1/31) |
