# Supplementary material for: Invasive Group A Streptococcus infections in children during the post-pandemic period: results from a multicenter study in Italy
Source: Ital J Pediatr. 2025 Nov 28;51:312. doi: 10.1186/s13052-025-02103-7 (PMC12661756; doi:10.1186/s13052-025-02103-7)
Supplement: Supplementary file 2 — Supplementary material 2 [file 13052_2025_2103_MOESM2_ESM.docx]

**Table S2:** Univariate analysis for factors associated with discharge with death or sequalae.

|  | **Univariate analysis** |  |  |  |
| --- | --- | --- | --- | --- |
| **Study population characteristics** | **n/N** | **OR** | **95% CI** | ***p*** |
| Male  Female | 9/48 (47.4%)  10/27 (52.6%) | 1  2.549 | 0.878-7.399 | 0.085 |
| Age   - 0-2 - 3-10 - >10 | 4/15 (26.7%)  13/52 (25.0%)  2/8 (25.0%) | 1  0.917  0.917 | 0.249-3.381  0.128-6.556 | 0.896  0.931 |
| Birthplace   - Italy - Another country | 18/66 (27.3%)  1/9 (11.1%) | 1  0.333 | 0.039-2.857 | 0.316 |
| Comorbidities   - No - Yes   Type of comorbidities   - Others - Neurologic - No - Respiratory | 15/62 (24.2%)  4/13 (30.8%)  1/3 (33.3%)  2/6 (33.3%)  15/63 (23.8%)  1/3 (33.3%) | 1  1.600  1  1  0.625  1 | 0.422-6.067  0.053-18.915  0.053-7.385  0.034-29.807 | 0.489  1  0.709  1 |
| Antibiotics in the previous month   - No - Yes - Missing | 12/50 (24.0%)  6/19 (31.6%)  1/6 (16.7%) | 1  1.462  0.633 | 0.456-4.685  0.067-5.967 | 0.523  0.690 |
| NSAIDs in the previous 15 days   - No - Yes - Missing | 7/41 (17.1%)  11/26 (42.3%)  1/8 (12.5%) | 1  3.562  0.694 | 1.156-10.979  0.073-6.568 | **0.027**  0.750 |
| Fever   - No - Yes | 0/3 (0.0%)  19/72 (26.4%) | omitted |  |  |
| Rash   - No - Yes | 13/56 (23.2%)  6/19 (31.6%) | 1  1.527 | 0.484-4.817 | 0.471 |
| On palate petechiae   - No - Yes | 17/69 (24.6%)  2/6 (33.3%) | 1  1.529 | 0.257-9.101 | 0.641 |
| Pain   - No - Yes   Types of pain   - Abdominal - Other - Osteo-articular - Cervical pain/headache - Sore throat - No - Earache | 5/28 (17.9%)  14/47 (29.8%)  4/8 (50.0%)  2/3 (66.7%)  4/16 (25.0%)  1/4 (25.0%)  2/10 (20.0%)  5/28 (17.9%)  1/6 (16.7%) | 1  1.952  1  2.000  0.333  0.333  0.250  0.217  0.200 | 0.617-6.173  0.125-31.975  0.056-1.995  0.023-4.736  0.031-1.999  0.040-1.178  0.016-2.575 | 0.255  0.624  0.229  0.417  0.191  0.077  0.217 |
| Upper airways infections*   - No - Yes | 10/39 (25.6%)  9/36 (25.0%) | 1  0.967 | 0.341-2.740 | 0.949 |
| Osteomyelitis and Septic Arthritis   - No - Yes | 17/62 (27.4%)  2/13 (15.4%) | 1  0.481 | 0.097-2.400 | 0.372 |
| Meningitidis   - No - Yes | 17/68 (25.0%)  2/7 (28.6%) | 1  1.200 | 0.213-6.764 | 0.836 |
| Pharyngitis   - No - Yes | 13/42 (30.9%)  6/33 (18.2%) | 1  0.496 | 0.165-1.490 | 0.211 |
| Cutaneous localization   - No - Yes | 15/65 (23.1%)  4/10 (40.0%) | 1  0.800 | 0.227-2.814 | 0.728 |
| Sepsis and septic shock   - No - Yes | 10/43 (23.3%)  9/32 (28.1%) | 1  1.291 | 0.454-3.676 | 0.632 |
| Transfer to PICU   - No - Yes | 5/41 (12.2%)  14/34 (41.2%) | 1  5.040 | 1.583-16.049 | **0.006** |
| First admission department   - Directly in PICU - No PICU - Transfer to PICU | 5/19 (26.3%)  5/41 (12.2%)  9/15 (60.0%) | 1  0.389  4.200 | 0.097-1.553  0.983-17.950 | 0.181  **0.053** |
| White Blood Count at admission:   - <4000/mm^3^ - 4000-20000/mm^3^ - >20000/mm^3^   WBC≥20000/mm^3^   - No - Yes   WBC<4000/mm^3^   - No - Yes | 2/6 (33.3%)  13/51 (25.5%)  4/18 (22.2%)  15/57 (26.3%)  4/18 (22.2%)  17/69 (24.6%)  2/6 (33.3%) | 1  0.648  0.571  1  0.800  1  1.529 | 0.112-4.183  0.075-4.345  0.227-2.814  0.257-9.101 | 0.681  0.589  0.728  0.641 |
| Neutrophils   - ≤ 10000/mm^3^ - >10000/mm^3^ | 10/39 (25.6%)  9/36 (25.0%) | 1  0.967 | 0.341-2.740 | 0.949 |
| C-reactive protein (CRP)   - CRP≤15.9 mg/dl - CRP 16.0-29.9 mg/dl - CRP>30.0 mg/dl | 8/37 (21.6%)  9/27 (33.3%)  2/11 (18.2%) | 1  1.813  0.806 | 0.592-5.551  0.144-4.501 | 0.298  0.805 |
| Positive Procalcitonin   - PCT≤0.5 ng/dl - PCT>0.5 ng/dl - Missing | 3/11 (27.3%)  15/49 (30.6%)  1/15 (6.7%) | 1  1.176  0.190 | 0.273-5.063  0.017-2.151 | 0.827  0.180 |
| Positive GAS blood culture or polymerase chain reaction (PCR)   - No - Yes | 11/48 (22.9%)  8/27 (29.6%) | 1  1.416 | 0.488-4.111 | 0.522 |
| Positive GAS culture or PCR on other sites   - No - Yes - Missing | 9/33 (27.3%)  10/26 (38.5%)  0/16 (0.0%) | 1  1.667  omitted | 0.555-5.010 | 0.363 |
| Viral coinfections   - No - Yes | 15/55 (27.3%)  4/20 (20.0%) | 1  0.667 | 0.192-2.318 | 0.524 |
| Bacterial coinfections   - No - Yes - *S. aureus* infections | 12/57 (21.1%)  4/12 (33.3%)  3/6 (50.0%) | 1  1.875  3.750 | 0.482-7.296  0.670-20.991 | 0.365  0.133 |
| Surgery   - No - Surgery - Drainage | 6/38 (15.8%)  4/10 (40.0%)  9/27 (33.3%) | 1  3.556  2.667 | 0.765-16.528  0.817-8.708 | 0.106  0.104 |
| Intravenous immunoglobulin (IVIG) use:   - No - Yes | 14/64 (21.9%)  5/11 (45.5%) | 1  2.976 | 0.790-11.214 | 0.107 |
| Steroids during hospitalization   - No - Yes | 11/49 (22.5%)  8/26 (30.8%) | 1  1.535 | 0.527-4.474 | 0.432 |
| Time between diagnosis and anti-toxin antibiotics start   - In the first 2 days - Over 2 days (≥2 days) | 11/33 (33.3%)  8/42 (19.1%) | 1  0.471 | 0.527-4.474 | 0.432 |
| Severe GAS infections  iGAS | 3/31 (9.7%)  16/44 (36.4%) | 1  5.333 | 1.397-20.363 | **0.014** |

*In Upper airways infections are included: acute mastoiditis, retropharyngeal abscesses, lymphadenopathies, and lymphadenitis.
