# Supplementary material for: Invasive Group A Streptococcus infections in children during the post-pandemic period: results from a multicenter study in Italy
Source: Ital J Pediatr. 2025 Nov 28;51:312. doi: 10.1186/s13052-025-02103-7 (PMC12661756; doi:10.1186/s13052-025-02103-7)
Supplement: Supplementary file 3 — Supplementary material 3 [file 13052_2025_2103_MOESM3_ESM.docx]

**Table S3:** Univariate analysis for factors associated with severe GAS infection

|  | **Univariate analysis** |  |  |  |
| --- | --- | --- | --- | --- |
| **Study population characteristics** | **n/N** | **OR** | **95% CI** | ***p*** |
| Male  Female | 23/48 (47.9%)  8/27 (29.6%) | 1  0,457 | 0.168-1.245 | 0.126 |
| Age   - 0-2 - 3-10 - >10 | 6/15 (40%)  23/52 (44.2%)  2/8 (25%) | 1  1.189  0.5 | 0.369-3.829  0.07-3.358 | 0.770  0.475 |
| Birthplace   - Italy - Another country | 27/66 (43.5%)  4/9 (55.6%) | 1  0,666 | 0.181-2.445 | 0.540 |
| Comorbidities   - No - Yes   Type of comorbidities   - Others - Neurologic - No - Respiratory | 27/62 (43,5%)  4/13 (30,8%)  1/3 (33,3%)  1/6 (16,7%)  27/63 (42,9%)  2/3 (66,7%) | 1  0,666  1  0,4  1,5  4 | 0.181-2.445  0.015-10.016  0.129-17.413  0.134-119.22 | 0.5  0.577  0.745  0.423 |
| Antibiotics in the previous month   - No - Yes - Missing | 22/50 (44.0%)  8/19 (42.1%)  1/6 (16.7%) | 1  0.925  0.254 | 0.318-2.693  0.02-2.340 | 0.887  0.226 |
| NSAIDs in the previous 15 days   - No - Yes - Missing | 20/41 (48.8%)  8/26 (30.8%)  3/8 (37.5%) | 1  0.466  0.63 | 0.165-1.312  0.132-2.988 | 0.148  0.560 |
| Fever   - No - Yes | 2/3 (66.7%)  29/72 (40.3%) | 1  0.337 | 0.029-3.892 | 0.38 |
| Rash   - No - Yes | 23/56 (42.9%)  8/19 (52.6%) | 1  1.04 | 0.363-2.996 | 0.936 |
| On palate petechiae   - No - Yes | 29/69 (42%)  2/6 (33.3%) | 1  0.689 | 0.118-4.022 | 0.679 |
| Pain   - No - Yes   Types of pain   - Abdominal - Other - Osteo-articular - Cervical pain/headache - Pharyngeal - No - Earache | 11/28 (39.3%)  20/47 (42.6%)  1/8 (12.5%)  1/3 (33.5%)  4/16 (25%)  2/4 (50.0%)  9/10 (90.0%)  11/28 (39.3%)  3/6 (50.0%) | 1  1.144  1  3.5  2.333  7  63  4.529  7 | 0.441-2.971  0.440-0.144  0.485-0.215  0.183-0.397  0.005-3.322  0.183-0.487  0.148-0.501 | 0.781  0.440  0.485  0.183  **0.005**  0.189  0.148 |
| Osteomyelitis and Septic Arthritis   - No - Yes | 28/62 (45.2%)  3/13 (23.1%) | 1  0.364 | 0.09-1.45 | 0.15 |
| Meningitidis   - No - Yes | 30/68 (44.1%)  1/7 (14.3%) | 1  0.211 | 0.02-1.849 | 0.160 |
| Pharyngitis   - No - Yes | 13/42 (31%)  18/33 (54.5%) | 1  2.676 | 1.038-6.903 | **0.041** |
| Sepsis and septic shock   - No - Yes | 24/43 (55.8%)  7/32 (21.9%) | 1  0.221 | 0.07-0.622 | **0.004** |
| White Blood Count:   - <4000/mm^3^ - 4000-20000/mm^3^ - >20000/mm^3^   WBC≥20000/mm^3^   - No - Yes   WBC<4000/mm^3^   - No - Yes | 1/6 (16.7%)  22/51 (43.1%)  8/18 (44.4%)  23/57 (40.4%)  8/18 (44.4%)  30/69 (43.5%)  1/6 (16.7%) | 1  3.79  4  1  1.182  1  0.26 | 0.413-34.833  0.385-41.511  0.405-3.446  0.028-2.344 | 0.239  0.246  0.758  0.229 |
| C-reactive protein (CRP)   - CRP≤15.9 mg/dl - CRP 16.0-29.9 mg/dl - CRP ≥30.0 mg/dl | 21/37 (56.8%)  6/27 (22.2%)  4/11 (36.4%) | 1  0.21  0.43 | 0.07-0.664  0.108-1.748 | **0.007**  0.240 |
| Positive Procalcitonin   - PCT≤0.5 ng/dl - PCT>0.5 ng/dl - Missing | 7/11 (63.6%)  13/49 (26.5%)  11/15 (73.3%) | 1  0.206  1.571 | 0.02-0.05  0.59-0.293 | **0.02**  0.597 |
