# Supplementary material for: Invasive Group A Streptococcus infections in children during the post-pandemic period: results from a multicenter study in Italy
Source: Ital J Pediatr. 2025 Nov 28;51:312. doi: 10.1186/s13052-025-02103-7 (PMC12661756; doi:10.1186/s13052-025-02103-7)
Supplement: Supplementary file 4 — Supplementary material 4 [file 13052_2025_2103_MOESM4_ESM.docx]

**Table S4:** Distribution of enrolled patients across study centers

| **Hospital** | **n. of patients**  **(N=75)** | **Male** | **female** | **Age**  **<2 y*** | **Age**  **2-10 y*** | **Age**  **>10 y*** |
| --- | --- | --- | --- | --- | --- | --- |
| **Salesi Children Hospital** | 3 (4%) | 2 | 1 | 0 | 1 | 2 |
| **Department of Medical and surgical sciences, Alma Mater Studiorum** | 3 (4%) | 2 | 1 | 3 | 0 | 0 |
| **ASST Spedali Civili of Brescia,** | 5 (6.6%) | 3 | 2 | 0 | 5 | 0 |
| **AOU Meyer, Florence** | 19 (25.3%) | 14 | 5 | 4 | 15 | 0 |
| **Fondazione IRCCS Ca' Granda Ospedale Maggiore Policlinico, SC Pediatria Pneumoinfettivologia, Milano** | 10 (13.3%) | 7 | 3 | 3 | 7 | 0 |
| **University Hospital Policlinico Federico II** | 3 (4%) | 0 | 3 | 0 | 1 | 2 |
| **Regina Margherita Children's Hospital,** | 10 (13.3%) | 6 | 4 | 2 | 7 | 1 |
| **Bambino Gesù Children's Hospital, IRCCS, Rome,** | 12 (16%) | 6 | 6 | 2 | 8 | 2 |
| **Division of Paediatric Infectious Disease, "G. Di Cristina" Hospital, ARNAS Civico Di Cristina Benfratelli, Palermo,** | 2 (2.6%) | 1 | 1 | 0 | 1 | 1 |
| **Fondazione Policlinico Universitario A. Gemelli IRCCS, Rome, Italy.** | 2 (2.6%) | 2 | 0 | 1 | 1 | 0 |
| **Department of Maternal, Infantile and Urological Sciences, Sapienza University of Rome, Umberto I** | 3 (4%) | 3 | 0 | 0 | 3 | 0 |
| **University Hospital of Udine** | 3 (4%) | 2 | 1 | 0 | 3 | 0 |
| **Total** |  | 48 (64%) | 27 (36%) | 15 (20%) | 52 (69,4%) | 8 (10,6%) |

*Age 2< years, 2-10 years, >10 years
